# Misleading localization by ^18^F-fluorocholine PET/CT in familial hypocalciuric hypercalcemia type-3: a case report

**DOI:** 10.1186/s12902-021-00683-z

**Published:** 2021-01-26

**Authors:** Noha N Mukhtar, Mohei El-Din M Abouzied, Mohammed H Alqahtani, Muhammad M Hammami

**Affiliations:** 1 Department of Medicine , King Faisal Specialist Hospital and Research Center, Riyadh, Saudi Arabia; 2 Department of Radiology, King Faisal Specialist Hospital, Riyadh, Saudi Arabia; 3 Department of Cyclotron and Radiopharmaceuticals, Alfaisal University, Riyadh, Saudi Arabia; 4 Department of Clinical Studies and Empirical Ethics, King Faisal Specialist Hospital and Research Centre, P O Box # 3354 (MBC 03), 11211 Riyadh, Saudi Arabia; 5 College of Medicine, Alfaisal University, Riyadh, Saudi Arabia

**Keywords:** ^18^F-fluorocholine positron emission tomography/ computed tomography, Primary hyperparathyroidism, Familial hypocalciuric hypercalcemia type-3, False positive localization

## Abstract

**Background:**

Familial hypocalciuric hypercalcemia (FHH) is a heterogeneous autosomal-dominant disorder of calcium hemostasis that may be difficult to distinguish clinically from mild primary hyperparathyroidism. Loss-of-function mutations mainly involving Arg15 residue of the adaptor-related protein complex 2, sigma subunit 1 (AP2S1) cause a rarer, more recently recognized form of FHH, FFH type-3. Recently, ^18^F-fluorocholine positron emission tomography/computed tomography (FCH-PET/CT) showed superior sensitivity to conventional imaging in localizing parathyroid adenomas. We report a new FFH type-3 patient who underwent unnecessary parathyroidectomy in association with misleading FCH-PET/CT imaging.

**Case presentation:**

A 29-year old woman was initially evaluated for parathyroid hormone (PTH)-dependent hypercalcemia in 2013. Medical history was positive only for chronic constipation and malaise with no personal or family history of hypercalcemia, kidney stones, or neck surgery. Over seven years, serum calcium level was 2.51–2.89 mmol/L with concomitant PTH level of 58.7–94.8 mmol/L. Serum phosphate levels were in the low/low normal range. Serum creatinine and magnesium levels were normal. 25-hydroxy vitamin D level was 13 nmol/L. 24-hour urine calcium level was 1.92 mmol/day but increased to 6.99 mmol/day after treatment with cholecalciferol 1000 IU daily. Bone mineral density and renal ultrasound were normal. Parathyroid ultrasound showed two hypoechoic nodules inferior to the left and right thyroid lobes; however, ^99m^technitium-sestamibi scans (2013, 2016, 2018) were negative. FCH-PET/CT (2019) showed focal uptake co-localizing with the nodule inferior to the left thyroid lobe. The patient underwent left inferior parathyroidectomy and pathology was consistent with parathyroid hyperplasia. However, postoperatively, serum calcium and PTH levels remained elevated and FCH-PET/CT and ultrasound showed persistence of the uptake/nodule. Whole exome sequencing showed Arg15Cys mutation in the AP2S1 gene characteristic of FHH type-3.

**Conclusions:**

In this new case of FHH type-3, FCH-PET/CT failed to localize to the hyperplastic parathyroid glands and localized instead to apparently a lymph node. This, together with increased urinary calcium after vitamin D supplementation, led to unnecessary parathyroidectomy. Given the increasingly lower cost of genetic testing and the cost of follow up and unnecessary surgery, it may prudent to include genetic testing for FHH early on in patients with mild PTH-dependent hypercalcemia.

## Background

Familial hypocalciuric hypercalcemia (FHH), a rare, generally benign, autosomal-dominant disorder of calcium hemostasis, is characterized by increased serum calcium level, non-suppressed parathyroid hormone level, and disproportionately low urinary calcium excretion [1–4].

Loss-of-function mutations in the calcium-sensing receptor (CaSR) gene (FHH type-1) [1], guanine nucleotide-binding protein subunit alpha 11(GNA11) gene (FHH type-2) [5, 6], and adaptor-related protein complex 2 sigma subunit 1 (AP2S1) gene (FHH type-3) [7–10] are responsible for most but not all FHH cases [11, 12]. The three genes are involved in circulating calcium signaling to the parathyroid glands and renal tubules, GNA11 mediated the action of CaSR and AP2S1 is important for clathrin-coated vesicle-mediated endocytosis of the CaSR [3].

FHH type-3 accounts for about 5 % of FHH cases and 13–22 % of CaSR mutation-negative cases [7, 10]. Affected individuals commonly harbor heterozygous germline mutations in Arg15 residue (Arg15His, Arg15Cys, and Arg15Leu); however, other residues may be also involved [9]. Unlike other FHH patients, patients with FHH type-3 may have symptomatic hypercalcemia [8, 11], reduced bone mineral density [13], cognitive impairment and behavioral disorders [3, 8, 12] and pancreatitis and chondrocalcinosis [1] Further, FHH type-3 has been associated with significantly higher serum calcium and magnesium levels and reduced fractional excretion of calcium compared with FHH type-1 [8].

In the absence of family history, it is clinically difficult to distinguish FHH from mild primary hyperparathyroidism (PHPT), a much more common disorder [14]. Such distinction is important to avoid unsuccessful and usually unnecessary surgical intervention.

Recently, ^18^F-fluorocholine positron emission tomography/computed tomography (FCH-PET/CT) has shown superior sensitivity in localizing parathyroid adenomas than conventional morphological and functional imaging [15–18]. FCH-PET/CT has been recommended when conventional imaging is negative or discordant [19–24] and also as first line imaging [25]. However, FCH-PET/CT specificity for parathyroid tissue (vs. other neck tissues) and its sensitivity in visualising hyperplastic parathyroid glands of FHH that are usually smaller than parathyroid adenomas, are not well known.

We report a new patient with FHH type-3 that underwent unnecessary parathyroidectomy in association with misleading FCH-PET/CT imaging.

## Case presentation

A 29-year old woman was initially evaluated for PTH-dependent hypercalcemia in 2013 when she was 22 years old. Apart from chronic constipation and malaise, her clinical evaluation was non-contributory; with no history of nausea, vomiting, abdominal pain, polyuria, or bone pain. She was not on drugs that could contribute to hypercalcemia. Her past medical history was unremarkable and negative for fracture, pancreatitis, kidney stone, and hypercalcemia; however, she did not have a documented calcium level measurement before. Family history was also negative for hypercalcemia, kidney stones, and neck surgery. Past surgical history was notable for nasal septoplasty in 2010 and scoliosis corrective surgery in 2016 (posterior spinal fusion and instrumentation). She had a body mass index (BMI) of 24 Kg/m^2^ and normal blood pressure. Physical examination was unremarkable.

Biochemical and hormonal investigations were performed at the Pathology and Laboratory Department, King Faisal Specialist Hospital and Research Center, Riyadh, Saudi Arabia. As shown in Table 1, serum calcium level was always high-normal, but never > 2.9 mmol/L. Associated parathyroid hormone (PTH) (electrochemiluminescense immunoassay, cobas e 80 immunoassay analyzer) levels were in the upper normal-mildly elevated range. Serum phosphate levels were in the low/low normal range. Serum magnesium level and renal function were normal. 25-hydroxy vitamin D level (electrochemiluminescense binding assay, cobas e 80 immunoassay analyzer) was initially low at 13 nmol/L and increased after treatment with cholecalciferol 1000 IU daily. Spot urine calcium/creatinine ratio was within the normal range (˂0.39) and lower than the hypercalciuric range (˃0.56). 24-hour urine calcium level was initially low at 1.92 mmol/day and increased on one occasion to 6.99 mmol/day. However, calcium to creatinine clearance ratio (CCCR), calculated based on 24-h urine collection, was < 0.01 (0.008 and 0.007). Bone mineral density (BMD) was normal for age with Z scores at lumbar spine, distal radius, and femoral neck of -1.1, 1.5, and − 0.1, respectively. Renal ultrasound (US) was unremarkable.

**Table 1:** Biochemical and hormonal investigations

	Dec 2013	Aug 2014	Sept 2015	Sept 2016	May 2017	April 2018	Dec 2018	Mar 2019	July 2019		Sept 2019	Mar 2020
									**Pre**	**Post**		
Serum calcium, mmol/L (NL, 2.1–2.6)	2.67	2.68	2.71	2.67	2.76	2.69	2.89	2.87	2.51	2.57	2.70	2.75
PTH, ng/l (NR, 15–65)	71.6	73	94.8	70.8	85.5	58.7	70.4	63.7		58.9	67.6	
25-hydroxy vitamin D, nmol/L (NL, > 75)			13	46	26	49	28.2	41.7			45	
Serum phosphate, mmol/L (NL, 0.9–1.5)	0.94	0.79	1.14	0.91	0.87	0.67	0.83	0.69	0.93	1.02	0.81	0.84
Serum magnesium, mmol/L (NL, 0.7-1)	0.89	0.88	0.87	0.84	0.91	0.85	0.89	0.87	0.86	0.84	0.84	0.87
Serum creatinine, umol/L (NL, 46–69)			58		56		54	46	52		52	57
24-h urine calcium, mmol/day (NL, 2.5–7.5)	1.92	2.74		2.32		2.83	3.93	6.99			3.19	3.35
Urine volume (L)	1.56	1.27		1.7		1.83	2.37	1.78			0.77	1.66
24-h urine creatinine, mmol/day (NL, 6–15)								13.6				10.2
CCCR								0.008				0.007
Spot urine calcium, mmol/L	1.23	2.16		3.14	3.99	1.55	1.66	3.93			4.15	2.02
Spot urine creatinine, mmol/L								11				6
Spot urine calcium/creatinine ratio (NL, ˂0.39)^a^								0.36				0.34

*NL* normal range, *PTH* parathyroid hormone, *CCCR* calcium creatinine clearance ratio. Pre and Post refer to date immediately before and after parathyroidectomy. ^a^Calculated as calcium (mmol/L)/creatinine (mmol/L), hypercalciuric range is > 0.56

**Fig. 1:**
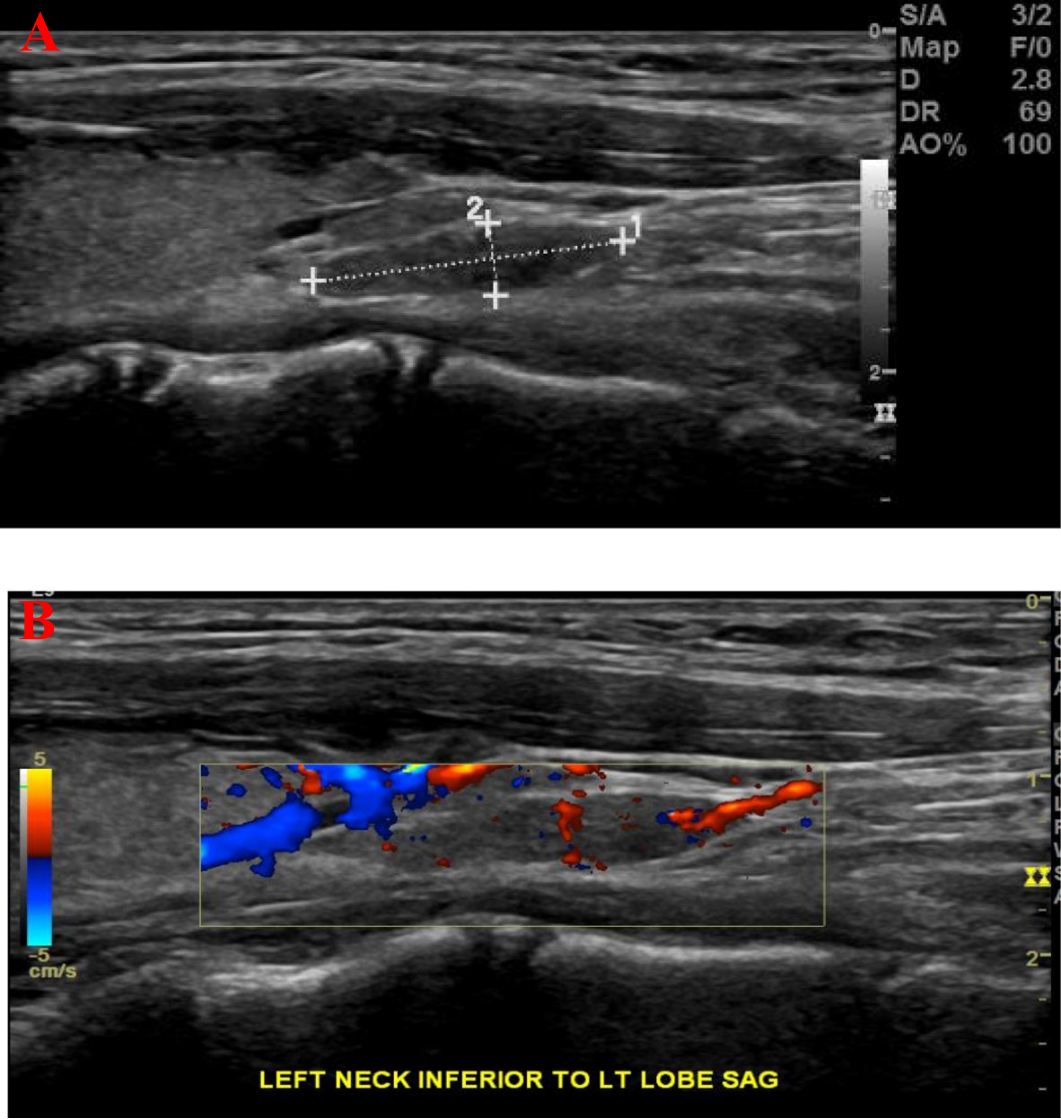
Parathyroid US. Sagittal view (**a** and **b**) showing an approximately 1.0 cm vascular hypoechoic nodule inferior to the left thyroid lobe

**Fig. 2:**
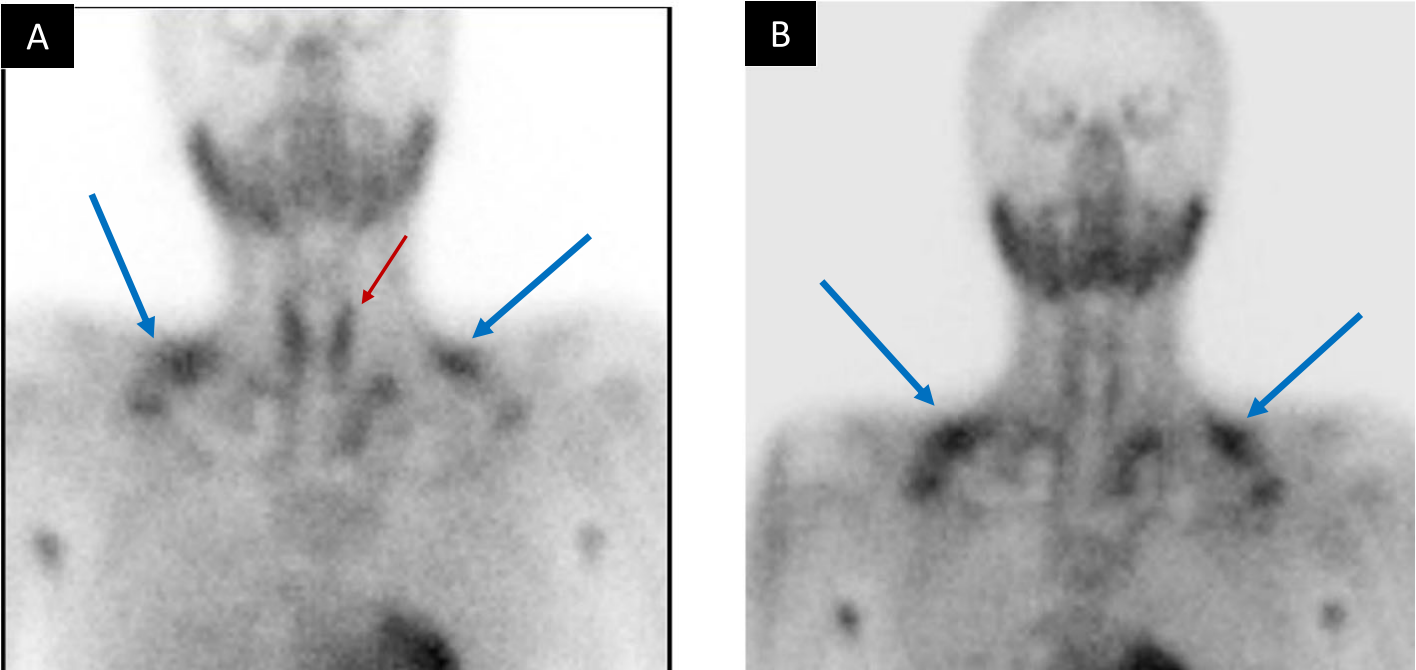
^99m^Technetium-sestamibi dual phase scan. **a**, anterior view twenty minutes after tracer administration showing homogeneous tracer distribution within the thyroid gland (red arrow) and normal variant brown fat uptake (blue arrows). **b**, 2-h delayed anterior view showing complete wash out of the tracer from both thyroid lobes, persistent brown fat uptake (blue arrows), and no abnormal tracer activity in the thyroid bed or elsewhere in the head, neck, and the mediastinum to qualify for parathyroid adenoma

**Fig. 3:**
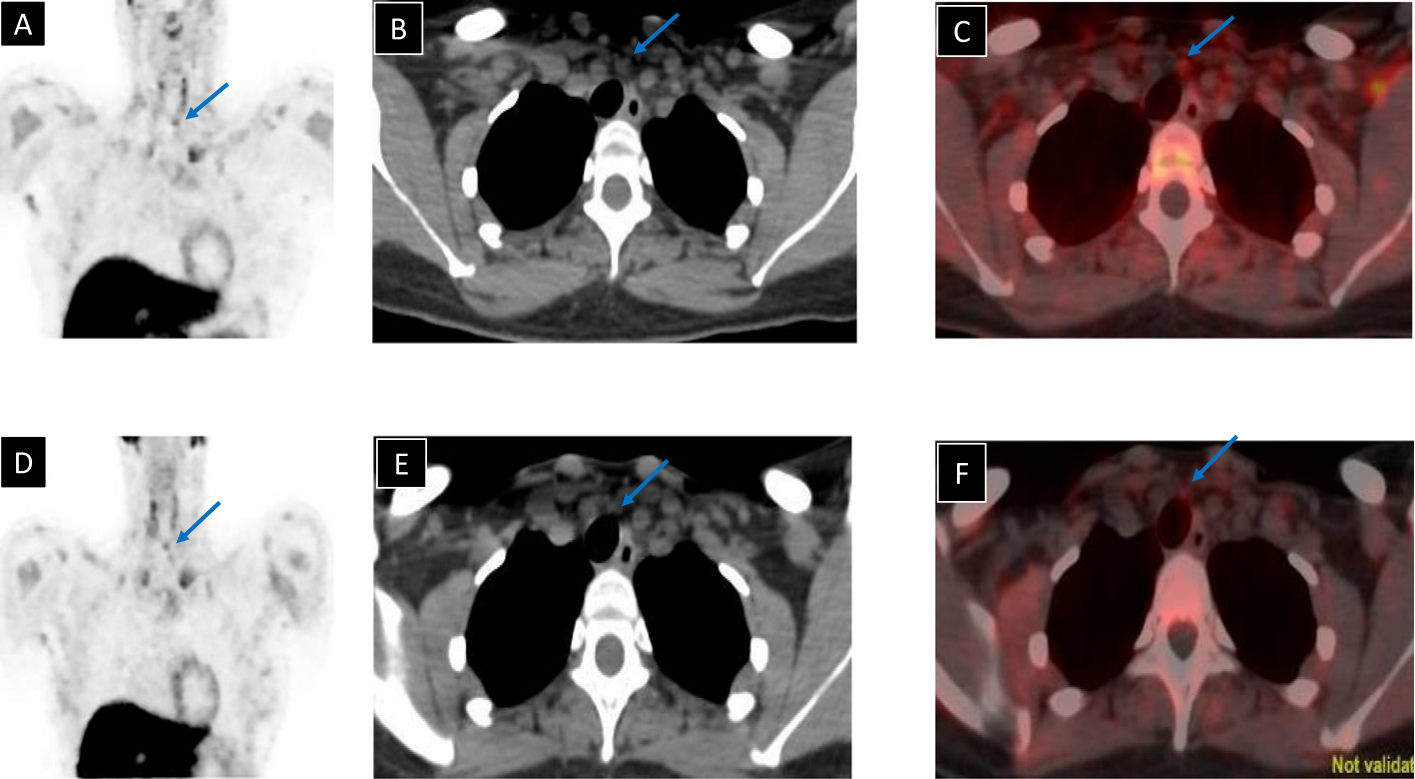
^18^F-fluoroholine PET/CT. Images were obtained before (**a**, **b**, and **c**) and after (**d**, **e**, and **f**) left inferior parathyroidectomy. Coronal PET (**a** and **d**), axial CT (**b** and **e**), and fused PET/CT (**c** and **f**) images show choline-avid lesion inferior to the lower pole of the left thyroid lobe (blue arrows) that persisted after parathyroidectomy

Parathyroid US in 2013 showed an oval-shaped hypoechoic soft tissue lesion (10 × 3 × 3 mm) posterior to the right thyroid lobe and a similar lesion (4, x 10 × 3 mm) posterior to the left thyroid lobe (Fig. 1a). The two lesions were vascular (Fig. 1b) and showed no clear fatty hila. A follow up parathyroid US (2016) did not report features suggestive of parathyroid adenoma. ^99m^Technitium-sestamibi (MIBI) scans (2013, 2016, and 2018) were negative (Fig. 2). FCH-PET/CT (May 2019) revealed abnormal focal choline uptake inferior to the left thyroid lobe that corresponded to few millimeter soft tissue density in the same location (Fig. 3a-c). FCH-PET/CT was performed per standard protocol. Briefly, after a minimum of 4 h fast, 4.0 MBq/ Kg of ^18^F-FCH were administered via intravenous injection and PET/CT examination was performed 60 minutes later. The PET/CT system has an axial field of view of 60 cm per bed position and an in-plane spatial resolution of 7 mm. The system acquires the CT first, followed by PET. After examination, CT and PET data sets can be viewed separately or in a fused mode on a commercially available computer workstation (Xeleris, GE healthcare, Milwaukee, WI, USA). Whole-body PET/CT with a field of view from the mandible to the carina was obtained. Data were acquired in a cranio-caudal direction with the patient in the supine position with a headrest and arms along the body, using a standardized breathing protocol.

Because of persistently high serum calcium, an increase in 24-h urine calcium to 6.99 mmol/day, and the FCH-PET/CT finding, the patient underwent an uncomplicated left inferior parathyroidectomy in July 2019. Pathology revealed tan smooth glistening soft tissue weighing 0.12 grams and measuring 1.2 × 0.7 × 0.3 cm with a histology consistent with parathyroid lipoadenoma or hyperplasia.

After surgery, serum calcium and PTH levels remained elevated, 24-h urine calcium was low, FCH-PET/CT (Oct 2019) showed a persistent mild, focal, tracer-avid soft tissue thickening inferior to the left thyroid lobe, suspicious for a parathyroid adenoma (Fig. 3d-f), and thyroid US showed persistence of the previously seen hypoechoic nodules inferior to the left and right thyroid lobes.

Whole exome sequencing (Baylor Genetics, Texas, USA) showed a previously reported heterozygous Arg15Cys (c.43 C˃T, p.R15C, chr19: 47,349,360) pathogenic variant in the AP2S1 gene, characteristic of FHH type-3. No other likely-pathogenic variants or variants of unknown clinical significance related to the clinical phenotype were detected.

### Discussion and conclusions

Initially, the patient had a clinical picture consistent with either mild PHPT or FHH; mild hypercalcemia with high normal/mildly elevated PTH, low urinary calcium but with concomitant vitamin D deficiency, no end organ damage, negative family history, no previous documentation of normal serum calcium level, and negative MIBI scan. However, an increase in 24-h urine calcium to 6.99 mmol/day after vitamin D treatment and a positive FCH-PET/CT scan that co-localized with a vascular lesion on US, inferior to the left thyroid lobe, led to unnecessary parathyroidectomy as the patient was later confirmed to have FHH type-3 by whole exome sequencing. As expected, the patient continued to have PTH-dependent hypercalcemia; however, unexpectedly, the target lesion persisted on follow up US and FCH-PET/CT. The 24-h urine calcium was misleading as was the FCH-PET/CT that instead of showing uptake in the four hyperplastic parathyroid glands, showed uptake in what appeared to be a lymph node.

### Calcium excretion

The three genes that may be mutated in FHH regulate not only PTH secretion but also renal calcium reabsorption. Hypercalcemia via normal CaSR (which is expressed throughout the kidney) reduces renal calcium reabsorption, mainly in the thick ascending limb of Henle’s loop. This mechanism is intact in PHPT but impaired in FHH [3, 11]. Hence, determination of urinary calcium excretion may help distinguish PHPT from FHH. This can be accomplished by spot urine calcium/creatinine ratio, 24-hour urine calcium, or CCCR. Our patient spot urine calcium/creatinine ratio was within the normal range. Although mean/median values of fasting urine calcium/creatinine ratios are higher in PHPT than FHH, most of the individual values overlap [4] and occasional FHH families may be hyercalciuric [26]. Further, urinary calcium/creatinine ratios should be interpreted with caution as vitamin D therapy may raise urinary creatinine level in addition to urinary calcium level [27]. Similarly, 24-h urinary calcium may be normal in FHH patients as seen in our patient and previously reported in another FHH type-3 patient [28]. CCCR using a separating point of 0.01 %-0.02 %, may be most useful; a CCCR less than 0.01 % has a sensitivity of 0.80 and specificity of 0.88 for FHH [29]. However, because PHPT is much more common, most patients with CCCR values near the 0.01 cut-off will have PHPT [30]. Our patient had a CCCR of 0.007/0.008.

### FCH-PET/CT sensitivity and specificity

Recently, surgical strategy for parathyroidectomy changed from bilateral cervical exploration to minimally invasive surgery, increasing the demand for accurate preoperative imaging. FCH PET/CT has been increasingly gaining acceptance as the best imaging modality [15–25]. Compared to conventional functional imaging, FCH-PET/CT does not only have superior sensitivity, but also better spatial resolution, lower radiation exposure, and shorter study time [15, 20, 21].

A recent systematic review (23 articles, 1112 patients) compared FCH-PET/CT with conventional morphological and functional imaging in patients with biochemical hyperparathyroidism and found that FCH-PET/CT sensitivity ranged from 58 %-100 % which may be related to type of conventional imaging, patient population, and FCH-PET/CT protocol [18]. More recent studies found FCH-PET/CT sensitivity of 91 % (84/92 lesions) when defining lesions with both positive and inconclusive FCH uptakes as positive [19], 94 % in 101 patients with PTH-dependent hypercalcemia and negative or discordant conventional imaging (vs. 45 % and 44 % for MIBI scan and US, respectively) [20], 92 % in 103 patients (vs. 39–56 % for conventional scintigraphy) [21], and 62 % 47 patients after inconclusive first line imaging including US and subtraction scan [22]. Previous meta-analyses showed pooled sensitivity of 90 % (8 studies, 272 patients) [15] and of 95 % on per-patient analysis and 92 % on per-lesion analysis **(**14 studies, 517 patients) [16]. Finally, a retrospective study of 50 PHPT patients (55 glands) with negative/discordant first-line imaging (MIBI scan and US) and histology with ≥ 50 % perioperative decrease in PTH level as gold standard, FCH-PET/CT had 93 % and 88 % sensitivity on per patient and per gland analysis, respectively, which was better than that of four-dimensional contrast-enhanced computed tomography (4D-CT) and similar to integrated FCH-PET/CT/4D-CT [31], a systematic review (16 studies, 619 patients) concluded that FCH-PET/CT is indicated when results of first-line tests are negative or discordant [17], and a recent retrospective study recommended FCH-PET/CT as first line imaging [25].

Nevertheless, sensitivity of FCH-PET/CT for multiple functioning parathyroid gland is less well documented and appears to be lower, which may be due to smaller size and lighter weight in multiple functioning glands. It was 78 % in 9 patients [20], and 88 % in 14 patients (4 with 2 adenomas and 10 with 31 hyperplastic glands) [21].

In addition, FCH-PET/CT specificity is not well known. The 2018 meta-analysis by Kim et al. showed a pooled specificity of 94 % [15] and in the recent systematic review by Evangelista et al., specificity ranged from 12.5 % (per lesion) to 100 % [18]. More recent studies showed a false positive rate of 8.7 % in 84 patients when defining lesions with both positive and inconclusive uptake as positive [19], 1 % in 103 patients [21], and 2 % in 105 patients [20]. Several causes of false positive FCH-PET/CT uptake have been reported, including ganglioneuroma and thyroid remnants, [31] well differentiated thyroid cancer and inflammatory lymph nodes [20], histologically classified thyroid tissue [21], thyroid nodules and normal reactive or metastatic lymph nodes [16], thyroid gland uptake [24], and thymoma in patient with familial primary hyperparathyroidism [32]. Choline is an important precursor for phospholipids biosynthesis. Increased uptake may be related to accelerated phosphatidylcholine turnover, upregulation of phospholipid-dependent choline kinase activity, or cholinergic autocrine loop upregulation and increased expression of choline transporters [15, 33–35].

To our knowledge, this is the first reported case of FHH type-3 in Saudi Arabia. Aashiq et al. recently reported an FFH type-3 case in a 4-year-oldboy with developmental and speech delay from United Arab Emirates who has Arg15His mutation [36]. Our patient appeared to be cognitively normal similar to the kindred reported by Wong et al. who also had Arg15Cys [28], suggesting a genotype-phenotype relation. However, no difference in phenotype was reported among the three genotypes of FFH type-3 in 19 patients (10 Cys, 5 His, 4 Leu) [4]. Our patient is also the first reported case of FHH where FCH-PET/CT failed to localize to hyperplastic parathyroid glands and localized instead to what appears to be a lymph node. Finally, it is interesting that our patient had scoliosis that required surgical intervention. The CaSR is expressed in chondrocytes and bone cells and experiments in CaSR knockout mice suggest that the CaSR plays a role in the embryonic development of the skeleton development and bone formation [37–39].

FHH frequently present considerable diagnostic challenge. PHPT is much more common than FHH and thus most patients with CCCR values near the 0.01 cut-off will have PHPT [30]. Further, although FHH is characterized with positive family history and mild hypermagnesemia [5, 40] these may be absent as demonstrated in the current case. Sporadically occurring new mutations may be found in 15–30 % of new FHH cases [3]. It is estimated that 9–23 % of patients with PTH-dependent hypercalcemia who underwent failed neck exploration may have FHH [2]. Furthermore, as seen in our patient, 15–20 % of patients with FHH may have a mildly elevated PTH concentration, especially in those with FHH type-3 [12].

A pro-FHH (stands for protect FHH patients) scoring system was developed for hypercalcemic patients with a PTH level within the normal range. It takes into account calcium, PTH, and serum osteocalcin levels and calcium-to-creatinine clearance ratio, and was reported to have higher accuracy than CCCR and 100 % specificity for PHPT [41]. Further, contrast enhanced US has been recently proposed to help differentiating intrathyroid parathyroid adenoma from thyroid nodules based on extrathyroidal blood supply of the former [42]. Furthermore, routine genetic testing has been recommended for patients with no clear surgical target and non-diagnostic CCCR [43] or when urine calcium excretion is inappropriate for serum calcium level, especially in younger patients [3, 44].

## Conclusions

In the current case, FCH-PET/CT failed to localize hyperplastic parathyroid glands and localized instead to apparently a lymph node. This, together with increased urinary calcium after vitamin D supplementation, lead to unnecessary parathyroidectomy. Given the increasingly reduced cost of genetic testing and the cost of follow up and unnecessary surgery, it may prudent to include genetic testing for FHH early on in patients with mild non-progressive PTH-dependent hypercalcemia despite negative family history and even positive localization. However, since about 30 % of FHH cases remain unclassified, it is likely that not all genes able to cause FHH are currently known, and a negative genetic test may not conclusively exclude FHH [41].

## Data Availability

All data generated or analyzed during this study are included in the published article.

## Abbreviations

AP2S1: Adaptor-related protein complex 2 sigma subunit 1
BMI: Body mass index
CaSR: Calcium-sensing receptor gene
CCCR: Calcium to creatinine clearance ratio
CT: Computed tomography
FCH-PET/CT: ^18^F-fluorocholinepositron emission tomography/computed tomography
FHH: Familial hypocalcuric hypercalemia
GNA11: Guanine nucleotide-binding protein subunit alpha 11
PHPT: Primary hyoerparathyroidism
PTH: Parathyroid hormone
MIBI scan: ^99m^Technetium-sestamibi dual phase scan
US: Ultrasound
4D-CT: Four-dimensional computed tomography
